# Back to the Origin: Mechanisms of circRNA-Directed Regulation of Host Genes in Human Disease

**DOI:** 10.3390/ncrna10050049

**Published:** 2024-09-24

**Authors:** Haomiao Yuan, Xizhou Liao, Ding Hu, Dawei Guan, Meihui Tian

**Affiliations:** 1 Center of Forensic Investigation, China Medical University, No. 77 Puhe Road, Shenyang North New Area, Shenyang 110122, China; hmyuan@cmu.edu.cn; 2 Liaoning Province Key Laboratory of Forensic Bio-Evidence Science, No. 77 Puhe Road, Shenyang North New Area, Shenyang 110122, China; 3 Department of Forensic Pathology, School of Forensic Medicine, China Medical University, No. 77 Puhe Road, Shenyang North New Area, Shenyang 110122, China; 4 Department of Forensic Genetic and Biology, School of Forensic Medicine, China Medical University, No. 77 Puhe Road, Shenyang North New Area, Shenyang 110122, China; 2019342217@stu.cmu.edu.cn (X.L.); 2019341815@stu.cmu.edu.cn (D.H.)

**Keywords:** circular RNA, host gene, regulatory mechanism, RNA-binding proteins, ceRNA

## Abstract

Circular RNAs (circRNAs) have been shown to be pivotal regulators in various human diseases by participating in gene splicing, acting as microRNA (miRNA) sponges, interacting with RNA-binding proteins (RBPs), and translating into short peptides. As the back-splicing products of pre-mRNAs, many circRNAs can modulate the expression of their host genes through transcriptional, post-transcriptional, translational, and post-translational control via interaction with other molecules. This review provides a detailed summary of these regulatory mechanisms based on the class of molecules that they interact with, which encompass DNA, mRNA, miRNA, and RBPs. The co-expression of circRNAs with their parental gene productions (including linear counterparts and proteins) provides potential diagnostic biomarkers for multiple diseases. Meanwhile, the different regulatory mechanisms by which circRNAs act on their host genes via interaction with other molecules constitute complex regulatory networks, which also provide noticeable clues for therapeutic strategies against diseases. Future research should explore whether these proven mechanisms can play a similar role in other types of disease and clarify further details about the cross-talk between circRNAs and host genes. In addition, the regulatory relationship between circRNAs and their host genes in circRNA circularization, degradation, and cellular localization should receive further attention.

## 1. Introduction

Circular RNA (circRNA) is a type of non-coding RNA (ncRNA) formed by the back-splicing of linear precursor mRNA (pre-mRNA) [1]. Due to the absence of a cap structure in the 5′ end and a poly-A tail in the 3′ end, circRNAs are not easily degraded by nucleic acid exonucleases, which makes them more stable than mRNAs [2,3]. This enhanced stability may be responsible for the higher expression levels of some circRNAs compared with their linear counterparts [3,4]. CircRNAs also have incredible diversity and tissue-specific expression, with the most abundant expressions being found in the nervous system, heart, and tumor tissues [5,6,7,8].

Advances in high-throughput sequencing technologies and bioinformatics algorithms have revealed a growing number of circRNAs playing roles in the control of different diseases [1,6,7]. However, the functions of these circRNAs still need to be further explored. In several significant reviews, the biological functions of circRNA could be summarized as follows: regulating the effects of back-splicing itself on canonical splicing, acting as microRNA (miRNA) decoys or “sponges”, serving as scaffolds or sequesters of proteins, and translating themselves [1,9,10]. The interactions of circRNAs with other molecules, including DNA, RNA, and proteins, run through these biological functions, and play a crucial role in influencing transcription and splicing, controlling the stability and translation of mRNAs in the cytoplasm, disrupting signaling pathways, and acting as templates for translation in various biological and pathophysiological situations [11,12,13,14,15,16,17,18,19]. 

In 2017, Tan et al. performed deep RNA sequencing on ribosomal-depleted RNA that was isolated from the cardiomyocytes of different species/developmental stages [20]. The results revealed that among the thousands of cardiac-expressed circRNAs, the most highly expressed circRNAs were generally correlated with the abundance of their cognate linear RNA, which is consistent with a competition mechanism that can produce a high number of circRNAs and alter the gene expression in *cis* by competing with the production of linear gene products from the same locus. In contrast, the study by Siede et al. raised the objection that circRNAs, independently of host gene expression profiles, were more likely to be involved in disease processes [21].

Although the evidence that circRNA exerts *trans*-regulation by sequestering miRNA or autoregulatory protein levels is exceptional, a few studies also indicated their *trans*-regulation function in the parental protein or linear RNA levels. For example, researchers showed that Muscleblind (MBL)-derived circRNAs are key regulators of MBL by means of *cis*- and *trans*-acting mechanisms in the fly brain and eye [22]. In the fly brain, circMbl regulates MBL-C protein *in cis*, while in photoreceptors, circMbl2-4 regulates *in trans* MBL-O/P proteins. Moreover, circMbl has functions *in trans* that are related to locomotor behavior. Another example is CDR1 anti-sense (CDR1as), which acts as a *trans*-regulator of CDR1 mRNA through a mechanism that is regulated by AGO2- and miR-671-mediated cleavage in the brain [23]. All these studies suggest that circRNAs may participate in the progress of diseases by regulating their parental genes, at least from the perspective of the regulatory mechanism. 

Therefore, this paper focuses on the interactive function of circRNAs with other molecules, and mainly reviews the regulatory mechanisms of circRNAs in the transcription, post-transcription, translation, and post-translation of host genes in diseases, thus providing a new direction for future research on circRNAs. 

## 2. Interaction with Nucleic Acid Molecules

CircRNAs interact with other nucleic acid molecules, including DNA and RNA, and other non-coding RNA (ncRNA), and may play a critical role in regulating cellular physiology [24,25]. 

### 2.1. Binding to Host Gene DNA

#### 2.1.1. Binding to Host Gene’s Promoter

**Figure 1:**
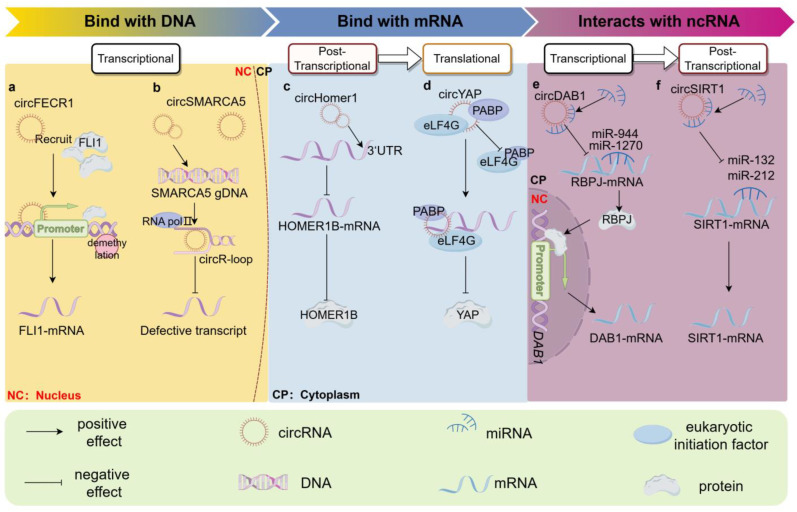
CircRNAs regulate host genes via interacting with nucleic acid molecules. Several circRNAs could interact with parental DNA sequences to regulate the transcription of their host genes. For example, FECR1 has the ability to attach to the *FLI1* promoter and regulate the hypomethylation of FLI1 DNAs (**a**), while circSMARCA5 can bind to its parental locus, leading to the formation of a circR-loop (circRNA: DNA hybrid), and transcriptional pausing at exon 15 of *SMARCA5* (**b**). CircRNAs interact with parental mRNA and could be divided by whether the binding site is in the translated regions. The upregulated circHOMER1 can correlate with the expression of *HOMER1B* mRNA by binding to the 3′-UTR of *HOMER1B* mRNA, thereby inhibiting the *HOMER1B* expression in post-transcriptional control (**c**). CircYAP could interact with eIF4G and PABP, along with *Yap1* mRNA, ultimately inhibiting *YAP1* translation in breast cancer (**d**); when circRNA and the host mRNA shared the same miRNAs directly, circRNAs could enhance mRNA stability as miRNA sponge, such as circSIRT (**f**). In addition, some circRNAs can indirectly regulate host gene expression by influencing other downstream transcription factor genes through the ceRNA mechanism, such as circDAB1. It could absorb miR-1270 and miR-944 in BMSCs to upregulate *RBPJ* expression. As a transcription factor, RBPJ could activate *DAB1* transcription, and facilitate cell proliferation and the osteogenic differentiation of BMSC (**e**).

Previous studies have shown that circRNAs could control the expression of their host gene by interacting with the host gene’s promoter, thereby influencing the processing of pre-mRNA. In transcriptional regulation, the promoter regions play a crucial role. The interaction between circRNAs and the promoter of the host gene can either enhance or inhibit the transcription of the host gene, a process that consistently requires RNA-binding proteins (RBPs). For example, Chen et al. [26] identified a novel circular RNA, FECR1 (hsa_circ_0000369), consisting of Friend leukemia virus integration 1 (*FLI1*) exons 4-2-3. FECR1 has the ability to attach to the *FLI1* promoter and recruits ten-eleven translocation 1 (TET1), an enzyme that plays a key role in DNA demethylation. The molecular constituents of FECR1/FLI1/TET1 employ a feedback loop to stimulate this through promoting DNA hypomethylation in the CpG islands of the promoter, ultimately controlling the metastasis of breast cancer (BC) cells (Figure 1a). Similarly, Liu et al. revealed that the LRCH3 gene-derived circLRCH3 (hsa_circ_0123265) was markedly upregulated in hypoxic pulmonary arterial smooth muscle cells (PASMCs). The upregulated circLCRH3 could bind to its host gene promoter region (from −618 to −606). This interaction induces an active chromatin state and the demethylation of DNA, consequently enhancing *LCRCH3* transcription and ultimately promoting the development of pulmonary hypertension [27].

#### 2.1.2. The Formation of the circR-Loop

In some cases, a strong binding interaction between the double-stranded DNA and circRNA strands could form a circR-loop (circRNA: DNA hybrid), which is a special three-stranded secondary nucleic acid structure [28]. Several studies have reported that circRNAs derived from exons can increase the occurrence of cognate exon-skipped alternative splicing, in turn driving different phenotypes, especially in some plants such as Arabidopsis [29]. Recently, Chen and colleagues proposed that the circR-loop might be involved in the degradation of circRNAs (such as ci-ankrd52), emphasizing again that the mechanism of the circR-loop was worthy of further investigation [30].

The formation of the circR-loop might play a distinct role in transcription processes based on the binding regions in its corresponding DNA locus. Xu et al. [14] found that the expression of circSMARCA5 (hsa_circ_0001445) was decreased in BC tissues compared with that of the adjacent normal tissues, while the expression of the host gene *SMARCA5* was upregulated. The overexpression of circSMARCA5 can bind to its parental locus, leading to the formation of circR-loop, resulting in transcriptional pausing at exon 15 of *SMARCA5*. The formation of a truncated non-functional protein cannot perform the function of SMARCA5 in DNA damage repair (Figure 1b). Additionally, a recently published study also proved that the hybrids of circRNAs and their cognate loci DNA could promote transcriptional pausing, proteasome inhibition, chromatin reorganization, and DNA breakage [19]. CircMLL (hsa_CIRCpedia_342868) is formed by the exons 9-10 of the mixed lineage leukemia (MLL) gene. It is abundant in infant leukemia and binds the MLL breakpoint cluster region. circR-loops formed by circMLL and 9-10 exons of MLL DNA loci are stronger than the traditional R loops involving cognate linear RNAs, suggesting that they could persist for longer and encourage genome instability. The overexpression of circMLL is able to promote the co-localization of its cognate locus with the other loci in the MLL recombinome in HEK293T cells, forming leukemic oncogenes.

### 2.2. Binding to Host Gene mRNA

To carry out their fine-tuning activities, circRNAs not only interact with chromatin but often directly pair with other RNA molecules. For instance, using the psoralen-crosslinking RNA pulldown technique, Manuel et al. detected RNA-RNA interactions (RRIs) in vivo and found that circRNA zinc finger protein 609 (circZNF609) could pair directly with a few mRNAs, including cytoskeleton-associated protein 5 (*CKAP5*) mRNA. The interaction between the circZNF609 and *CKAP5* mRNA favors the binding of the human antigen R (HuR) to the *CKAP5* mRNA, leading to a stabilized microtubule cytoskeleton and enhanced tumor cell proliferation [31].

#### 2.2.1. Interacting with Translated Region of Parental mRNA

In 2019, Wu et al. first reported that a *YAP1*-derived circRNA named circYAP (hsa_circ_0002320) has the ability to bind directly with its parental mRNA and inhibit the translation of its associated gene in BC [15]. Normally, eIF4G interacts with PABP to enhance the translation by promoting the circularization of *YAP1* mRNA. The excessive production of circYAP could interact with eIF4G and PABP, along with the *Yap1* mRNA, ultimately inhibiting *YAP1* translation initiation by disrupting the connection between PABP and eIF4G on the *YAP1* mRNA 5′-cap and poly (A) tail, respectively (Figure 1d). This study revealed an innovative molecular process and suggested a new function of circRNA in regulating host gene expression in diseases.

#### 2.2.2. Interacting with Untranslated Region of Parental mRNA

Using biotinylated DNA probes or CrossLinking Poly (A) Pulldown RNase R Sequencing (CLiPPR-seq) technology, the interaction between circRNAs and mRNAs has gradually been confirmed [25]. A typical instance is circHOMER1 (hsa_circ_0006916), which is derived from exons 2 to 5 of *HOMER1B*. CircHOMER1 is upregulated in the orbitofrontal cortex (OFC), which is negatively correlated with the expression of *HOMER1B* mRNA. By binding to the 3′-untranslated region (UTR) of the *HOMER1B* mRNA, the predicted neural Hu protein D (HuD)-binding sites could also be affected (Figure 1c). The interaction between the circHOMER1 and *HOMER1B* mRNA enhances OFC performance, decreases synaptic translation, and controls reversal learning by influencing the stability and localization of neuronal mRNAs [32]. Taken together, the data reveal an antagonistic interplay between circRNAs and their linear mRNA counterparts, which is important in brain function and diseases.

### 2.3. Acting as miRNA Decoys

#### 2.3.1. ceRNA Hypothesis and miRNA “Sponge”

The competitive endogenous RNA (ceRNA) hypothesis is proposed by Salmena et al. and provides a new model for gene expression regulation at the transcriptional and post-transcriptional levels [33]. In this model, multiple RNA transcripts, including long non-coding RNAs (lncRNAs), pseudogene transcripts, and circRNAs, with the same miRNA-binding sites, could be regarded as miRNA “sponges” that competitively sequester away the miRNA from its target sites (also known as miRNA response elements or MREs) [34]. It has been touted as a new paradigm to explain the complexities of pervasive transcription, while also being met with growing skepticism [35,36,37]. The main controversy over whether circRNAs could be considered as “sponges” was the question of target site abundance [37]. Some circRNAs that are recognized as miRNA “sponges” often contain dozens of miRNA-binding sites, such as circSRY-miR138 [38], CDR1as-miR7 [39], and circZNF91-miR-23b-3p [40]. However, many studies reported circRNAs with single or a few MREs and low expression levels as miRNA “sponges”, thus limiting their potency. Importantly, extremes like CDR1as that contains 73 miR-7-binding sites are very rare in physiological processes [39]. Nonetheless, during cell differentiation and in the context of malignant transformation (such as a malignant tumor), the expression of target molecules might change dramatically. Thus, in this part, we do not focus excessively on the miRNA “sponge” function of circRNAs (the binding of one circRNA with a variety/number of miRNAs). Correspondingly, the representative circRNAs that have been verified by commonly used methodologies [35,41] (e.g., bioinformatics analyses, correlation analysis among circRNA, miR and miR target gene expression levels, co-localization studies, manipulation of circRNA/miRNA abundance, direct validation of circRNA–miRNA binding, and luciferase reporter assays), which can prove the binding of miRNA and circRNA, are summarized. 

The regulation of the expression of host genes through the ceRNA mechanism can be divided into two main categories based on whether miRNA directly binds to circRNAs’ parental mRNA. 

#### 2.3.2. Direct Competition between circRNAs and mRNAs for miRNA Binding

Studies on circRNAs competing directly with their parental mRNAs for miRNAs via ceRNA mechanisms have mostly focused on tumors.

Since 2017, many circRNAs have been demonstrated to play tumor facilitator or suppressor roles in multiple cancers. As an example, Zeng et al. identified circVANGL1 (hsa_circ_0002623), which was derived from the back-splicing of exon 3-4 of *VANGL1* and was significantly upregulated in bladder cancer (BCa) tissues. Luciferase assays confirmed the interactions of miR-605-3p with circVANGL1 and *VANGL1* mRNA 3′-UTR, respectively. The increased circVANGL1 expression hinders the effectiveness of miR-605-3p’s regulation of *VANGL1 mRNA*, resulting in the upregulation of *VANGL1*, which, subsequently, contributes to the proliferation, migration, and invasion of tumors, as well as a decreased 5-year survival rate for patients [42]. Zhou et al. [43] identified that circENO1 (hsa_circ_0000013) and its host gene, Enolase 1 (*ENO1*), are upregulated in lung adenocarcinoma (LUAD) cells. Within LUAD, circENO1 has the ability to interact with miR-22-3p, leading to the enhancement of *ENO1* expression. As a glycolytic enzyme, *ENO1* is crucial in tumor growth by stimulating glycolysis in the Warburg effect. The same principles were also found by other researchers, such as with circKEAP1 (hsa_circ_0049271) and miR-141-3p. 

In addition, a single circRNA can act as one “sponge” for multiple miRNAs, thus co-regulating the parental gene [44]. For example, Jiang et al. noted an increase in the levels of the circXPO1 (hsa_circ_0001016) and Exportin 1 (*XPO1*) mRNAs, both of which were associated with a negative outlook for individuals diagnosed with osteosarcoma (OS). CircXPO1 acted as a sponge for miR-23a-3p, miR-23b-3p, miR-23c, and miR-130a-5p to control the expression of XPO1 in OS cells. Therefore, suppressing circXPO1 or *XPO1* expression might be a novel therapeutic strategy for OS [45]. These results indicated that circRNAs and their host gene may serve collectively as potential molecular biomarkers in the diagnostic, therapeutic, and prognostic targets for cancers. The details of the direct regulation of parental gene expression by circRNA via the ceRNA mechanism in tumors are shown in Table 1 [16,42,43,45,46,47,48,49,50,51,52,53,54,55,56,57,58,59,60,61,62,63,64,65,66,67,68,69,70,71].

This direct regulatory mechanism has also been reported in nononcologic diseases, such as osteoporosis, and vascular smooth muscle growth and differentiation. Huang et al. identified circYAP1 (hsa_circ_0024097) as a ceRNA that enhances *YAP1* expression by sequestering miR-376b-3p. An upregulated expression of *YAP1* triggered the activation of the Wnt/β-catenin pathway, leading to the enhancement of osteoblast differentiation in both bone marrow stem cells (BMSCs) and MC3T3-E1 cells [72]. In the vascular inflammatory response, Kong et al. revealed that circSITR1 (hsa_circ_0093887) participated in the vascular smooth muscle cell (VSMC) inflammatory response and neointimal hyperplasia. Sirtuins (SIRTs) are a family of histone deacetylases that have seven subtypes (SIRT1-7). The expression of the Sirtuin 1 gene (*SIRT1*) was found to regulate various cardiovascular diseases (CVDs) such as cardiac hypertrophy (CH), ischemia–reperfusion injury, and vasculitis [73]. By means of RIP, RNA in situ hybridization, and luciferase reporter assay, researchers demonstrated that circSIRT1 directly binds to miR-132/miR-212, and enhances the SIRT1 mRNA activity (Figure 1f) [74]. Analogously, the mutual regulation among circRNAs-miRNAs-host genes in VSMCs was also shown in abdominal aortic aneurysms. *CCDC66* is associated with the growth and apoptosis of VSMCs. CircCCDC66 (hsa_circ_0001312) was upregulated among the circular transcripts of *CCDC66* after Angiotensin II (Ang II) treatment in VSMCs. Mechanically, it targeted miR-342-3p to upregulate *CCDC66* and alleviated VSMC proliferation and loss [75]. 

The situation can be more complex when additional factors such as the expression abundance of circRNAs and the number of miRNA-binding sites are taken into account. For example, in myocardial infarction, sucrose nonfermenting 1-related kinase (*SNRK*) has the potential to increase the production of ATP, lower oxygen consumption, and enhance the efficiency of mitochondria. Wang et al. [76] found that *SNRK*-derived circSNRK (hsa_circ_0004089) is principally in the cytoplasm of cardiomyocytes and provides seven possible binding sites of miR-33. Their direct connection is supported by the dual-luciferase reporter gene, FISH assays, and an RIP of AGO2. In addition, dual-luciferase reporter gene assay and immunoblot also clarified that miR-33 could directly bind to *SNRK* 3′ UTR to suppress its translation. Considering the expression abundances of circSNRK to linear SNRK (2%) and the amplification effects of miR-33 to circSNRK (7:1), the reduction in circSNRK was dominated by the downregulated SNRK via miR-33. The consequence of this negative feedback is to constrain the SNRK protein at an appropriate level, which suggests that the function of circSNRK with SNRK in regulating energy metabolism in cardiomyocytes could be a potential therapeutic target for heart failure.

#### 2.3.3. Influencing Downstream Genes through the ceRNA Mechanism

In addition to their direct effects on host genes, some circRNAs can indirectly regulate host gene expression by influencing other downstream transcription factor genes through the ceRNA mechanism. As an example, Liu and colleagues discovered that circSTAT3 (hsa_circ_0043800) showed a notable increase in hepatoblastoma (HB) cells, leading to the enhanced proliferation, invasion, and migration of HB cells. *STAT3*, a signal transducer and activator of transcription genes, controls the activity of numerous genes in response to cellular signals and is crucial for regulating cell growth and programmed cell death, particularly in HB. As previously stated, circSTAT3 modulates *STAT3* expression by acting as a sponge for miR-29a/b/c-3p. Furthermore, the decrease in circSTAT3 levels also led to a significant reduction in *Gli2* expression through the shared miRNAs. As the transcription factor for *STAT3*, *Gli2* could enhance the STAT3 expression in HB cells, potentially introducing a new regulatory pathway for HB treatment [77]. 

The interplay between circRNAs and host genes, as well as other downstream transcription factor genes, was also observed in osteogenesis. Chia et al. [78] demonstrated that increasing the expression of circDAB1 (hsa_circ_0113689) promoted the growth and bone-forming ability of BMSCs while reducing circDAB1 had the opposite outcome. Although circDAB1 sponged multiple miRNAs and positively affected DAB1 transcription, there was no direct ceRNA axis between circDAB1 and DAB1. Following this, the scientists showed that circDAB1 absorbed miR-1270 and miR-944 in BMSCs to upregulate *RBPJ* expression, an important transcription factor that activates *DAB1* transcription through the Notch pathway. Enhanced interactions with the RBPJ-DAB1 promoter facilitate cell proliferation and osteogenic differentiation (Figure 1e). Zhao et al. [79] discovered that circNOTCH1 (hsa_circ_0089547), derived from *NOTCH1*, exhibits an increased expression in gastric cancer (GC) tissues and cells and can regulate *NOTCH1* expression. Further research demonstrated that although circNOTCH1 can bind to miR-449c-5p, the host gene *NOTCH1* was not the sponge target. Both the database and experimental findings suggested that *MYC* was the target and could interact with the *NOTCH1* promoter to control the progression of GC. The findings above show that circ-NOTCH1 enhances metastasis and stemness in GC through the regulation of the miR-449c-5p/MYC/NOTCH1 pathway, indicating that circNOTCH1 could serve as a potential therapeutic target for GC. Analogously, An et al. [80] showed that circLECRC (hsa_circ_0004140) blocks the advancement of CRC by regulating the circLECRC/miR-135b-5p/KLF4 pathway to prevent the excessive activation of cancer-causing YAP1 signaling in CRC. CircLECRC acts as a regulator to prevent the excessive activation of the host gene *YAP1*, indicating that circLECRC could be a valuable target for CRC treatment. 

Collectively, circular RNAs have the ability to indirectly control the expression of their host genes by influencing other downstream genes via absorbing miRNAs. This indirect regulatory role may enable circRNAs to carry out their fine-tuning activities in multiple diseases.

## 3. Binding to Proteins

CircRNAs’ capacity to engage with proteins, resulting in the binding, sequestering, or translocation of proteins to specific subcellular compartments, enables them to function as dynamic scaffolds that regulate protein–protein interactions [17]. By binding to RBPs, circRNAs may participate in regulating the host gene at the transcription, post-transcription, translation, and post-translation levels in multiple diseases. 

### 3.1. CircRNAs Affect the Transcription of Host Genes

Based on the mode of biogenesis and the location, circRNAs can be classified into exonic circular RNAs (ecircRNAs), intronic circular RNAs (ciRNAs), and exon–intron circular RNAs (EIciRNAs) [81]. Through different mechanisms, it has been verified that certain circRNAs stimulate or suppress the transcription of their host genes by recruiting proteins, thereby fulfilling their functions of advancing or impeding disease progression. 

#### 3.1.1. EcircRNAs in Parental Transcription Regulation

EcircRNAs primarily localize to the cytoplasm. In addition to adsorbing miRNAs, many ecircRNAs are also able to influence parental gene transcription by interacting with functional RBPs, which are common in tumors.

**Figure 2:**
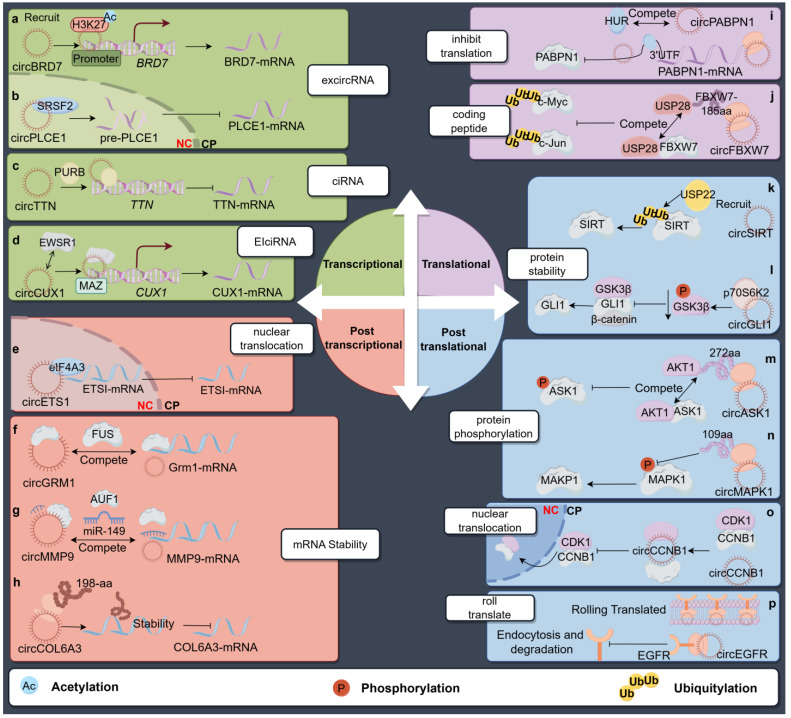
CircRNAs regulate host gene expression by binding with protein molecules in transcriptional, post-transcriptional, translational, and post-translational control. At the transcriptional level, ecircRNAs in different cellular locations might play various regulation functions. For example, cytoplasmic localized circBRD7 can recruit H3K27 acetylation in the promoter region of the BRD7, increase the transcriptional activation, and boost the expression of BRD in NPC (**a**). On the contrary, circPLCE1 inhibits the spliceosome assembly on pre-PLCE1 and hinders intron removal by interacting with the SRSF2 protein in the nucleus (**b**). ciRNA and EIciRNA (such as circTTN and circCUX1) could also bind to BRPs and thus interact with parental DNA sequences to regulate gene transcription (**c**,**d**). At the post-transcriptional level, circRNAs that are localized in the nucleus, such as circETS1, could bind to EIF4A3 to block the export of ETS1 mRNA from the nucleus (**e**), and the others in the cytoplasm, such as circGRM1, circMMP9, and circCOL6A3, could form a circRNA-RBP complex, associating with miRNAs or their translational peptide, resulting in the alteration of parental mRNA stability (**f**–**h**). At the translational level, certain circRNAs could hinder the binding of BRP to parental mRNA and impede the translation of host genes, such as circPABPN1 (**i**). Others like circFBXW7 have the ability to encode a short peptide, which could compete for the binding to USP28 with FBXW7, thus antagonizing the USP28-induced stabilization of c-Myc and c-Jun (**j**). At the post-translational level, circRNAs could bind or recruit RBPs, thus resulting in the ubiquitination or phosphorylation of the proteins (**k**–**n**). Moreover, circCCNB1 could regulate the nuclear translocation of CCNB1 by interacting with CDK1 (**o**). Furthermore, the rolling translation EGFR (rtEGFR) directly interacts with EGFR, weakening endocytosis and the degradation of EGFR proteins (**p**).

However, although rare, ecircRNA localization in the nucleus can also affect parental gene transcription through interactions with RBPs. For instance, SRSF2, a protein that is rich in serine and arginine, contains an RNA recognition motif (RRM) domain and often interacts with particular splicing enhancer sequences, serving as a broad splicing activator [82]. In CRC, circPLCE1 (hsa_circ_0019230) interacts with the SRSF2 protein, antagonizing the splicing of SRSF2-dependent phospholipase C epsilon 1 (*PLCE1*) pre-RNA. The inhibition of the spliceosome assembly on *pre-PLCE1* hinders intron removal during transcription by interacting with the SRSF2 protein, leading to the advancement of CRC (Figure 2b) [82,83].

#### 3.1.2. CiRNAs in Parental Transcription Inhibition

In contrast to ecircRNAs, ciRNAs are abundantly distributed in the nucleus with little enrichment for miRNA target sites [84]. However, researchers found that the knockdown of ciRNAs could lead to the reduced expression of their parent genes. In the case of ci-ankrd52 (derived from the second intron of *ANKRD52*), it primarily gathers at its parental gene transcription loci to interact with the elongating Pol II complex, ultimately enhancing transcription activity [84]. Similarly, ciRNA’s involvement in parental gene transcription has also been reported in diseases. TTN, the protein encoded by *Titin*, is the largest protein that is known to play essential roles in myogenesis. CircTTN originates from *Titin*’s intron 15, spanning 339 bases in full length. The increased expression of circTTN suppresses the proliferation and differentiation of myoblasts in mammals [85]. Ai et al. [86] demonstrated that circTTN is capable of enlisting the Pur-beta (*PURB*) protein, a transcription factor that hinders the proliferation and differentiation of myoblasts in C2C12 cells. The formation of heterotypic complexes negatively regulates the transcription of *Titin* (Figure 2c). 

#### 3.1.3. EIciRNAs in Parental Transcription Activation

Moreover, EIciRNAs can also affect parental gene expression at the transcriptional level by interacting with RBPs. Li et al. [87] found that circCUX1 (hsa_circ_0132813), encoded by CUT-like homeobox 1 (*CUX1*), exhibited high levels of expression in neuroblastoma (NB) and contributed to aerobic glycolysis and NB progression. The 393-nt circCUX1 consists of the exon 2 and partial intron 2 of *CUX1*. CircCUX1 interacts with EWS RNA-binding protein 1 (EWSR1) to enhance its binding with MYC-associated zinc finger protein (MAZ), leading to the increased transactivation of MAZ and the subsequent transcriptional changes in genes linked to tumor progression, including CUX1. These results suggested that targeting the circCUX1/EWSR1/MAZ axis could be a promising strategy for inhibiting aerobic glycolysis and slowing down the progression of neuroblastoma (Figure 2d). 

Overall, circRNAs could bind proteins to activate or inhibit the transcription of the host gene, which is a critical mechanism of circRNAs that are involved in disease progression.

### 3.2. CircRNA Affects Host Gene Expression in Post-Transcriptional Control

#### 3.2.1. Regulation in Parental mRNA Nuclear Translocation

According to the subcellular distribution of circRNAs, the mechanisms of regulating parental genes at the post-transcriptional level are also different. Mao et al. [88] discovered that hsa_circ_0004296 (derived from *ETS1*) was downregulated in the PCa tissue, blood, and urine of patients, indicating its potential as a noninvasive liquid biopsy marker for prostate cancer. Hsa_circ_0004296 is predominantly distributed in the nucleus in PCa cells. It can bind to EIF4A3 to block the export of the *ETS1* mRNA from the nucleus, leading to the suppression of *ETS1* gene expression at the post-transcriptional level and effectively suppressing the proliferation, migration, invasion, and epithelial–mesenchymal transition of PCa cells (Figure 2e).

#### 3.2.2. Regulation of Parental mRNA Stability

Unlike the degradation of the host gene mRNA through the ceRNA mechanism, the circRNAs that are localized in the cytoplasm affect parental gene expression, mainly by regulating the stability of the host gene mRNA through interactions with proteins. For example, in patients with atopic dermatitis and psoriasis, hsa_circ_0004287 is increased in peripheral blood mononuclear cells (PBMCs) and suppresses M1 macrophage activation in vitro. The overexpression of hsa_circ_0004287 in macrophages reduced skin inflammation in mice with conditions resembling atopic dermatitis and psoriasis. Mechanistically, hsa_circ_0004287 decreased the stability of its host gene metastasis-associated lung adenocarcinoma transcript 1 (*MALAT1*) by competitively binding to IGF2BP3 with MALAT1 in an m^6^A-dependent manner. Reduced *MALAT1* expression facilitated S100A8/S100A9 degradation through ubiquitination, consequently inhibiting p38/MAPK phosphorylation and inflammation mediated by macrophages [89]. Similarly, circGrm1 (mmu_circ_0001907) was upregulated in hypoxic pulmonary artery smooth muscle cells (PASMCs). Transcriptome sequencing indicated that Glutamate receptor 1 (*Grm1*) may be the target gene of circGrm1. It was found that circGrm1 can competitively bind to FUS, forming a circGrm1/FUS complex, consequently resulting in reduced mRNA stability and the expression of *Grm1*. Moreover, Grm1 can inhibit the Rap1 signaling pathway, accompanied by the activation of the Rap1/ERK signaling pathway, and inhibit the proliferation and migration of PASMCs, thereby inducing the occurrence of pulmonary arterial hypertension (Figure 2f) [90]. 

Some circRNAs promote the stability of the mRNA of the parental gene through recruiting some functional proteins with or without the assistance of miRNAs. Xia et al. [47] discovered circMMP9 (hsa_circ_0001162), which showed an increased expression in oral squamous cell carcinoma (OSCC) tissues, plasma cells, and cell lines, suggesting that it could serve as a valuable prognostic indicator similar to its host gene, *MMP9*. CircMMP9 functions by interacting with both AUF1 and miR-149 to prevent their inhibitory effects on *MMP9* 3′-UTR, ultimately leading to the increased stability of the *MMP9* mRNA and promoting metastasis in OSCC (Figure 2g). Zang et al. [49] found that circCCND1 (hsa_circ_0023303) was significantly upregulated in laryngeal squamous cell carcinoma (LSCC), which was strongly positively correlated with the *CCND1* mRNA expression level. In terms of mechanisms, circCCND1 functions as an effective sponge for miR-646 and alleviates the miR-646 inhibition of *CCND1* mRNA. Moreover, circCCND1 physically interacts with HuR to increase the binding of HuR to *CCND1* mRNA. The ability of circCCND1 to directly interact with HuR and miR-646 enhances the stability of *CCND1* mRNA, leading to increased *CCND1* expression and facilitating LSCC growth. 

In the mechanism of non-small-cell lung cancer (NSCLC), NSCLC cell growth is dependent on *NOTCH1*. As an m^6^A methyltransferase, methyltransferase-like 14 (*METTL14*) is a particularly important and well-known RBP that can decrease RNA stability through recruiting YTH m^6^A RNA-binding protein 2. CircNOTCH1 (hsa_circ_0089552) can compete with *NOTCH1* mRNA for METTL14 binding. Due to the lack of m^6^A modification by METTL14 on *NOTCH1* mRNA, *NOTCH1* mRNA is more stable and can much more easily undergo protein translation, thus promoting the growth of NSCLC [91].

Even more interestingly, the translation products of some circRNAs, which can act as RNA-binding proteins themselves, directly modulate the expression of parental genes at the post-transcriptional level. For example, as a tumor promoter, COL6A3, which is produced by the host gene *COL6A3*, plays a crucial role in the extracellular matrix and promotes tumor growth in CRC. Zhang et al. [92] clarified that *COL6A3* and *COL6A3*-derived hsa_circ_0006401 were both significantly upregulated in metastatic CRC tissues. Simultaneously, hsa_circ_0006401 can encode the 198-aa peptide, which is involved in the poly(A) mRNA decay process as an RNA-binding protein. The novel hsa_circ_0006401 peptide decreases the mRNA and protein levels of the host gene *COL6A3* by promoting *COL6A3* mRNA stabilization, thereby promoting CRC proliferation and metastasis (Figure 2h). Collectively, circRNAs can regulate the expression of their parental gene by promoting or inhibiting mRNA stability by binding RBPs.

### 3.3. CircRNA Affects Host Gene Expression in Translational Control

By enlisting proteins with roles in translation regulation, circRNAs potentially influence the expression of their host genes at the translational level. In human cervical carcinoma HeLa cells, Abdelmohsen et al. [93] demonstrated that circPABPN1 (previously known as hsa_circ_0031288) was a major target HuR. As an RBP, HuR has the ability to bind hundreds of mRNA and ncRNAs to affect the translation of multiple mRNAs, just as mentioned above [31,49]. Moreover, Yang et al. discovered that circHuR (hsa_circ_0049027), a circular RNA derived from 3 to 5 exons of HuR, was able to bind to CCHC-type zinc finger nucleic acid-binding protein (CNBP) and inhibited its interaction with the HuR promoter, leading to the decrease in HuR expression and the inhibition of gastric cancer advancement [94]. In this study, HuR had no impact on the levels of circPABPN1, but it notably hindered the binding of HuR to *PABPN1* mRNA and impeded the translation of *PABPN1* (Figure 2i). This groundbreaking regulatory model represents the initial instance of a circRNA influencing the translation of its corresponding mRNA through competition, resulting in decreased RBP availability.

In different circumstances, circRNAs control not just the synthesis, location, and degradation of proteins but also serve as scaffolds or attractors for proteins. They can even translate to short peptides that exhibit active biological functions to endorse their linear counterparts. In 2017, Zhang and colleagues [95] presented the pioneering discovery that circFBXW7 (hsa_circ_0001451) has the ability to encode a short peptide, FBXW7-185aa. This protein was found to suppress the proliferation of glioma cells and interfere with the cell cycle both in vitro and in vivo. The FBXW7 gene is widely known as a tumor-suppressive E3 ligase that is responsible for degrading key cellular regulators such as c-Myc, cyclin E1, c-Jun, and Notch1. As a tumor-suppressive decoy, FBXW-185aa inhibits USP28’s binding to FBXW7α, thus antagonizing the USP28-induced c-Myc stabilization. The study suggested that the circFBXW7-produced FBXW7-185aa could act as a form of “multiple safe assurances” in managing cellular growth by controlling the expression of host genes at the translational level (Figure 2j).

Overall, circRNAs could directly affect the translation processes of host genes or translate to short peptides to carry out indirect regulation in host genes at the translational level.

### 3.4. CircRNA Affects Host Gene Expression in Post-Translation Control

Some researchers also indicated that circRNAs might regulate the activity and degradation of host genes by recruiting RBPs to post-translationally modify the parental proteins, such as through phosphorylation, ubiquitination, and nuclear translocation.

#### 3.4.1. Regulation in Parental Protein Stability

Ubiquitination plays a vital role in numerous biological processes, such as cell survival, differentiation, and innate and adaptive immunity. CircRNAs may mediate the ubiquitination or deubiquitination of the parental gene by directly recruiting proteins or, alternatively, through the translation of functional short peptides. In Ang II-induced mouse CH models, Wang et al. proved the essential function of downregulated circSirt1 (mmu_circ_0002354) derived from exons 2 to 4 of *SIRT1*, which is highly homologous to hsa_circ_0093884. The stability of the Sirt1 protein was associated with the ubiquitination mediated by enzymes. Knocking down *Sirt1* could potentially counteract the impacts of upregulating circSirt1 and autophagy both in vitro and in vivo. The decreased expression of circSirt1 might elevate the expression of its parent gene at a post-transcriptional level through the sequestration of miR-3681-3p/miR-5195-3p (Figure 2k). Conversely, circSirt1 is able to stabilize the Sirt1 protein in hiPSC-CMs by enlisting USP22 to assist in the deubiquitination process, acting as a suppressor for CH [73]. In addition, functional short peptides derived from circRNA can also affect ubiquitination. As an illustration, Zhang and his colleagues [96] reported that circSHPRH (hsa_circ_0001649), a form of the SNF2 histone linker PHD RING helicase gene (*SHPRH*), can encode a novel protein. CircSHPRH utilizes overlapping genetic sequences to produce a “UGA” termination codon, leading to the synthesis of the 17 kDa SHPRH-146aa protein. SHPRH-146aa mechanically prevents the degradation of full-length SHPRH through the ubiquitin–proteasome pathway. Consequently, the stabilized SHPRH protein sequentially adds ubiquitin molecules to proliferating cell nuclear antigens as an E3 ligase, leading to the inhibition of cell growth and tumorigenesis. 

In addition to ubiquitination, circRNAs can also regulate the stability of parental proteins by binding to proteins at the post-translational level. In melanoma, circGLI1 (hsa_circ_0027247) is highly expressed in melanoma cells and has the potential to regulate migration, invasion, and angiogenesis. CircGLI1 annotated to the GLI family zinc finger 1 gene (*GLI1*) serves as the terminal effector molecule in Hedgehog signaling. The interaction between CircGLI1 and RPS6KB2 (specifically p70S6K2) can deactivate GSK3β by phosphorylating it at Ser9. This phosphorylation prevents GSK3β from binding to *GLI1* and β-catenin, leading to the inhibition of degradation and the increased expression of *GLI1* and β-catenin in melanoma (Figure 2l) [97]. This mechanism adds complexity to the regulation of both proteins and circRNAs, underscoring its potential significance in cellular physiology.

#### 3.4.2. Regulating Phosphorylation of Parental Gene 

The coding functions of circRNAs derived from certain critical kinases can participate in the regulation of the phosphorylation of the parental gene by competing with their parental proteins. Acting as a central signaling junction that triggers the JNK and p38 pathways to prompt apoptosis in gefitinib-resistant LUAD cells, *ASK1* serves as a stress-activated kinase [98]. In gefitinib-resistant LUAD cells, Wang et al. demonstrated that circASK1 (hsa_circ_0007798) was downregulated and could encode a novel polypeptide, ASK1-272aa. This type of short peptide is important for the activation of the ASK1/JNK/p38 pathway and mediates the chemical sensitivity induction of circASK1 in LUAD. In particular, ASK1-272aa competes with ASK1 to bind to Akt1, which opposes the phosphorylation and inactivation of ASK1 induced by Akt1, ultimately enhancing gefitinib-induced apoptosis and decreasing the resistance to gefitinib (Figure 2m). This discovery provides a new therapeutic target for overcoming gefitinib resistance in patients with LUAD [99].

Around the same time, a similar regulatory mechanism was also confirmed in GC. Jiang et al. [100] found that circMAPK1 (hsa_circ_0004872) encodes the novel protein MAPK1-109aa, functioning as a cancer suppressor by binding to MEK1 and preventing the phosphorylation of MAPK1, leading to the inhibition of MAPK1 activation and its related elements in the MAPK pathway (Figure 2n). 

#### 3.4.3. Regulation of Nuclear Translocation of the Parental Protein

One circRNA can also simultaneously bind to multiple functional proteins to regulate disease progression. For example, Yang’s team found that circCCNB1 (hsa_circ_0072758) was downregulated in BC. CircCNB1 originates from exon 4 and exon 5 of the cyclin B1 (*CCNB1*) gene, which is a regulator of cell mitosis [101]. The interaction between CircCCNB1 and H2AX prevents the binding of mutant p53, ultimately resulting in the apoptosis of cancer cells. Additionally, circCCNB1 can also bind with its host gene *CCNB1* and cyclin-dependent kinase 1 (CDK1) proteins, preventing the formation of the CCNB1-CDK1 complex and modulating the nuclear translocation of these two molecules, thereby influencing cell migration, invasion, and proliferation, and mouse survival (Figure 2o) [102]. 

#### 3.4.4. Rolling Translation of circRNAs

Various components are necessary for circRNA translation, such as internal ribosomal entry sites (IRESs), open reading frames (ORFs), and m^6^A modifications [96]. In theory, if stop signals are not present in every reading frame, some circRNAs may create endless open reading frames (iORFs), leading to the translation of lengthy repetitive proteins [103]. However, it was not until 2020 that Liu et al. [104] first demonstrated that endogenous circEGFR (hsa_circ_0080229) derived from the epidermal growth factor receptor (*EGFR*) gene contains an iORF. It encodes a new polymeric protein complex called the rolling translation EGFR (rtEGFR) in glioblastoma (GBM). EGFR amplification and/or mutation are present in 57% of the primary GBM cases, serving as both diagnostic and therapeutic markers. rtEGFR directly interacts with EGFR, continuously activating carcinogenic signals by maintaining the membrane localization of EGFR, thus weakening endocytosis and the degradation of EGFR proteins (Figure 2p). Depriving circEGFR clinically inhibits the tumorigenicity of brain tumor-initiating cells and enhances the efficacy of nimotuzumab in treating GBM. The study not only enhanced our comprehension of the molecular foundation behind a previously unidentified circRNA translation process, but it also proposed a novel method of the interaction between circRNAs and their host genes.

## 4. The Influence of Different Categories of circRNAs on Parental Gene Expression

Based on the number of studies summarized in this review, it seems that ecircRNAs are more advantageous, almost across all the levels of regulation in parental gene expressions. There may be three reasons for this phenomenon. Firstly, it is undeniable that ecircRNAs are more abundant and highly expressed than the other two types of circRNAs, and thus, they have also been more widely and thoroughly explored. Since they originated from the exon sequences related to splicing factors, ecircRNAs are more evolutionarily conserved between species [105]. These advantages in ecircRNAs’ expression patterns provide opportunities for animal experiments in vivo and demonstrate their significance in disease diagnosis and treatment. Several studies have demonstrated that ecircRNAs are likely to be transported by the nuclear export system or escape from nuclei during cell division, leading to an enrichment in the cytoplasm [76,89,105,106,107]. Cytosolic localization may also support the post-transcriptional function of ecircRNAs in parental gene regulation. Secondly, ecircRNAs harbor multiple miRNA-binding sites, of which some can interact with their linear counterparts. At present, most of these studies enhanced the target abundance by constructing overexpression vectors and rarely paid attention to their physiological expression levels. An understanding of such regulatory mechanisms with physiological states will help considerably in developing efficient diagnostic methods or even therapies. Furthermore, although non-coding sequences are also crucial in the regulation of gene expression, the sequences of the coding regions are more critical in determining phenotype and function, particularly as some of the ecircRNAs have coding functions themselves [108], by interacting with multiple molecules, or directly on host genes.

Although small in number, ciRNAs and EIciRNAs also play key roles in parental gene transcriptional regulation. Distinct from ecircRNAs, EIciRNAs are predominantly localized in the nucleus and are longer [109]. Analysis data from human organs indicated their higher expressions in the brain and testis than in the other tissues. In EIciRNAs, exons are circularized with “retained” introns, giving them the capacity to interact with U1 snRNP and thereby promoting the expression of their host genes (such as circEIF3J and circPAIP2) [110]. Similarly, ciRNAs are plentiful in the nucleus and show minimal presence of miRNA-binding sites. Thus, the binding to the RNA polymerase or transcription factor machinery mainly acts as a regulator of transcripts [84]. The knockdown of ciRNAs derived from the introns of *ANKRD52*, *MCM5*, and *SIRT7* could cause reductions in their parental mRNA expressions, suggesting a *cis*-regulatory role in their parental coding genes [84]. When ciRNAs bind to transcription factors, they can also play a *trans*-role in transcription (such as circTTN). It must be pointed out that although the mechanistic study of ciRNAs and EIciRNAs is more difficult than with ecircRNAs, it is also imperative.

## 5. Regulatory Network of circRNAs and Parental Genes as Potential Diagnostic and Therapeutic Biomarkers in Human Diseases

As an emerging type of non-coding RNA, circRNAs can be characterized by the following biological features: (1) high abundance [111]; (2) tissue-/disease-specific expression patterns [8]; (3) better stability compared with linear counterparts [4]; and (4) enrichment in exosomes [112]. These features lead to their ability to be potential biomarkers. The cross-talk between circRNAs and their genes has also revealed their co-expression as molecular targets in the diagnosis, treatment, and prognosis of human diseases [113]. For example, as mentioned above, circXPO1 and XPO1 could serve as an innovative treatment approach for OS [45], while targeting the circBRD7/BRD7 axis shows great potential as a strategy for the diagnosis and treatment of NPC in a clinical setting [106]. The therapeutic agents based on the co-regulation between circRNAs and their host genes, which target the circRNAs’ interactions with multiple molecules, have mainly focused on inhibiting the binding of RBPs or silencing RNA. Despite the advent of genetic engineering and circRNA vaccines, there are no drugs for clinical use for this treatment [114]. 

**Figure 3:**
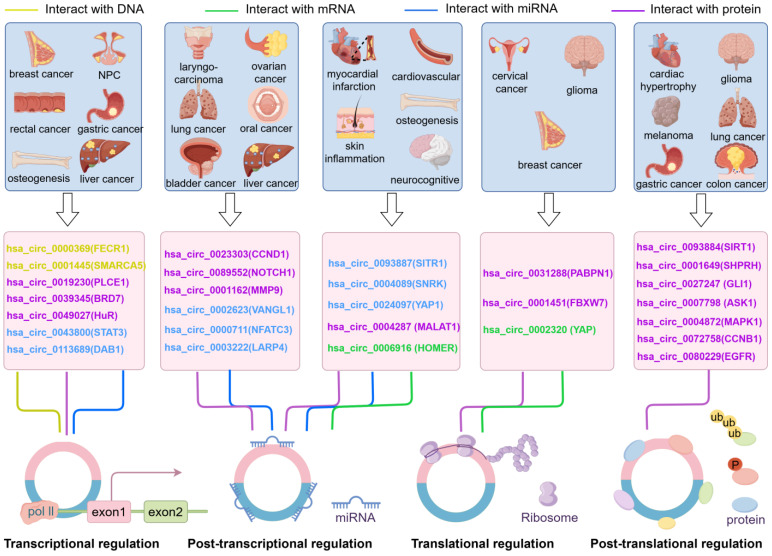
CircRNAs play crucial roles in the pathogenesis of numerous human diseases by modulating the expression of their host genes. In transcriptional regulation, circRNAs could interact with DNA, miRNAs, and proteins to fulfill their regulation in host genes. In the post-transcriptional, translational, and post-translational regulatory levels, circRNAs fulfill their parental gene regulatory functions in both neoplastic and non-neoplastic diseases mainly by adsorbing miRNAs or binding to proteins. The direct interactions of circRNAs with parental mRNAs are only mentioned in post-transcriptional and translational regulations.

The different regulatory mechanisms of circRNAs on their host gene through interacting with molecules make up a complex regulatory network. CircRNAs derived from different back-splicing actions of the same parental gene present dual or multiple functions in different diseases. For instance, in this review, three *YAP1*-derived circRNAs were reported. Although all these circRNAs are ecircRNAs, they show different regulation mechanisms on the parental genes by interacting with nucleic acid molecules: hsa_circ_0002320 has the ability to bind directly with its parental mRNA and inhibit its translation in BC [15]; and hsa_circ_0024097 [72] and circLECRC [80] act as ceRNAs to enhance or inhibit *YAP1* expression in osteoporosis and CRC. Similarly, hsa_circ_0089547 [79] and hsa_circ_0089552 [91] are derived from *NOTCH1*. Hsa_circ_0089547 can bind to miR-449c-5p, which targets MYC, and the upregulated MYC expression enhances its combination with the NOTCH1 promoter, thereby facilitating the transcription of *NOTCH*1 in gastric cancer [79]. Hsa_circ_0089552 can compete with *NOTCH1* mRNA for METTL14 binding, and promote the stability of *NOTCH1* mRNA at the post-transcriptional level in NSCLC [91]. In conclusion, circRNAs regulate the expression of their parental genes through multifarious mechanisms at the transcriptional, post-transcriptional, translational, and post-translational levels, which form a complex regulatory network in various human diseases (Figure 3).

In terms of expression, circRNAs with similar expression patterns of their host genes are more likely to participate in diseases by regulating parental genes. Taking post-transcriptional regulation through the competitive binding of the same miRNA as an example, an upregulated circRNA expression could adsorb more miRNAs, thus resulting in less binding to and cutting of the 3′-UTR of the mRNA and ultimately leading to the increased expression of the parental gene [16,61]. This process does not seem to be consistent with the competition between circRNA and pre-mRNA during the splicing process, which precisely illustrates the important function of circRNA in diseases. The synergistic role of circRNAs and their host genes form a positive feedback loop to play a critical role (such as FECR1 in breast cancer), while other circRNAs antagonize the functions of their host genes and play negative feedback roles (such as circSNRK in myocardial infarction).

## 6. Conclusions, Limitations, and Future Perspectives

Current studies on the regulatory relationship between circRNAs and their host genes mainly focus on ceRNA mechanisms and pay insufficient attention to physiological expression abundance [33]. In addition to acting as miRNA “sponges”, circRNAs can also regulate the expression of parental genes by directly binding to their DNA or mRNA transcripts. More importantly, the protein-binding functions of circRNAs also play crucial roles [9]. Future studies should explore whether these proven mechanisms can play similar roles in other types of diseases and clarify the cross-talk between circRNA and host genes through their interaction with other molecules in more detail. In addition, the regulatory relationship between circRNAs and their host genes in circRNA circularization, degradation, and cellular localization should be given further attention. 

In any case, the mechanism by which circRNAs regulate their parental genes provides new targets and directions for the diagnosis, treatment, and prognosis of various human diseases.

## Figures and Tables

**Table 1:** The direct regulation of parental gene expression by circRNA via the ceRNA mechanism in tumors.

Disease	CircRNA ID ^1^	Exp.circR ^2^	Host Gene	Exp.mR ^3^	Rel. miRNA	Val. Meth ^4^	MREs	Ref.
BGM	hsa_circ_0008278	down	*EPB41L5*	down	miR-19a	i, v, vi	1	[46]
OSCC	hsa_circ_0001162	up	*MMP9*	up	miR-149	v, vi	1	[47]
LSCC	hsa_circ_0036722	down	*RHCG*	down	miR-1248	i, vi	2	[48]
	hsa_circ_0023303	up	*CCND1*	up	miR-646	i, v, vi	3	[49]
BC	hsa_circ_0101187	up	*YY1*	up	miR-769-3p	ii, v, vi	1	[50]
	hsa_circ_0005239	up	*GFRA1*	up	miR-34a	i, ii, iii, iv, v, vi	2	[51]
	hsa_circ_0089105	down	*ASS1*	down	miR-4443	i, iii, v	1	[52]
	hsa_circ_0001451	down	*FBXW7*	down	miR-197-3p	i, ii, v, vi	2	[16]
LCa	hsa_circ_0000013	up	*ENO1*	up	miR-22-3p	i, ii, iii, iv, v, vi	2	[43]
	hsa_circ_0049271	down	*KEAP1*	down	miR-141-3p	i, ii, v, vi	1	[53]
	hsa_circ_0018414	down	*DKK1*	down	miR-6807-3p	i, ii, v, vi	1	[54]
	has_circ_0006404	down	*FOXO3*	down	miR-155	i, ii, v	1	[55]
	hsa_circ_0094342	down	*PTEN*	down	miR-155/miR-330-3p	i, ii, iv, v, vi	1	[56]
BCa	hsa_circ_0002623	up	*VANGL1*	up	miR-605-3p	i, ii, iii, iv, v, vi	1	[42]
	hsa_circ_0003323	up	*APP*	up	miR-186-5p	i, ii, v, vi	1	[57]
	hsa_circ_0084171	up	*FNTA*	up	miR-370-3p	i, ii, iv, v, vi	2	[58]
HCC	hsa_circ_0000711	down	*NFATC3*	down	miR-548i	i, iv, v, vi	1	[59]
	hsa_circ_0039411	up	*MMP2*	up	miR-136-5p	i, iv, v, vi	1	[60]
	hsa_circ_0010090	up	*FBLIM1*	up	miR-346	i, iv, v, vi	1	[61]
	hsa_circ_0003141	up	*UBAP2*	up	miR-1827	i, iii, iv, vi	1	[62]
	hsa_circ_0001649	down	*SHPRH*	down	miR-127-5p/miR-612/ miR-4688	ii, iv, v, vi	1	[63]
GC	hsa_circ_0006401	up	*COL6A3*	up	miR-3064-5p	i, v, vi	1	[64]
	hsa_circ_0069765	up	*KIT*	up	miR-142-5p/miR-144-3p/miR-485-3p	i, ii	N/A	[65]
	hsa_circ_0084097	up	*PLAT*	up	miR-142-5p/miR-144-3p/miR-485-3p	i, ii	N/A	[65]
	hsa_circ_0079741	up	*ETV1*	up	miR-142-5p/miR-144-3p/miR-485-3p	i, ii, iii	N/A	[65]
PC	hsa_circ_0000069	up	*STIL*	up	miR-144	i, ii, iii, iv, v, vi	1	[66]
PCa	hsa_circ_0004907	up	*ZEB*	up	miR-141-3p	ii, iv, v, vi	1	[67]
OC	hsa_circ_0003222	down	*LARP4*	down	miR-513b-5p	i, v, vi	1	[68]
	hsa_circ_0002346	up	*CRIM1*	up	miR-145-5p	ii, iv, vi	1	[69]
CC	hsa_circ_0033550	up	*AKT1*	up	miR-942-5p	i, ii, iv, v, vi	1	[70]
	hsa_circ_0004214	up	*AMOTL1*	up	miR-485-5p	i, ii, iv, v, vi	1	[71]
OS	hsa_circ_0001016	up	*XPO1*	up	miR-23a-3p/miR-23b-3p/ miR-23c/miR-130a-5p	i, ii, v, vi	1 *	[45]

^1^ circRNA ID comes from circBase (http://circbase.org/); ^2^ Exp.circR: the expression pattern of circRNA; ^3^ Exp.mR: the expression pattern of mRNA; ^4^ Val. Meth: the validation method reference for the Olesen study as followed: (i) bioinformatics analyses; (ii) correlation analysis among circRNA, miR, and miR target gene expression levels; (iii) co-localization studies; (iv) the manipulation of circRNA/miR abundance; (v) the direct validation of circRNA–miR binding; and (vi) luciferase reporter assays to validate circRNA–miR interactions; abbreviations involved in the table: glioblastoma (BGM); oral squamous cell carcinoma (OSCC); laryngeal squamous cell carcinoma (LSCC); breast cancer (BC); lung cancer; bladder cancer (BCa); hepatocellular carcinoma (HCC); gastric cancer (GC); pancreatic cancer (PC); prostate cancer (PCa); ovarian cancer (OC); cervical cancer (CC); osteosarcoma (OS); not available (N/A); * these four miRNA shared the same one MREs.
